# Kangbainian Lotion Ameliorates Vulvovaginal Candidiasis in Mice by Inhibiting the Growth of Fluconazole-Resistant *Candida albicans* and the Dectin-1 Signaling Pathway Activation

**DOI:** 10.3389/fphar.2021.816290

**Published:** 2022-01-20

**Authors:** Zewei Chen, Tengshuo Luo, Fengke Huang, Fuzhen Yang, Wenting Luo, Guanfeng Chen, Mengfei Cao, Fengyun Wang, Jun Zhang

**Affiliations:** ^1^ School of Pharmaceutical Sciences, Guangzhou University of Chinese Medicine, Guangzhou, China; ^2^ School of Chemistry and Molecular Engineering, East China Normal University, Shanghai, China; ^3^ The Second Clinical College, Guangzhou University of Chinese Medicine, Guangzhou, China; ^4^ School of Chinese Materia Medica, Guangdong Pharmaceutical University, Guangzhou, China

**Keywords:** kangbainian lotion, fluconazole-resistant, vulvovaginal candidiasis, *Candida albicans*, dectin-1 signaling pathway

## Abstract

Vulvovaginal candidiasis (VVC) is an infectious disease caused by *Candida* species, which affects millions of women worldwide every year. The resistance to available antifungal drugs for clinical treatment is a growing problem. The treatment of refractory VVC caused by azole-resistant *Candida* is still facing challenges. However, research on new antifungal drugs is progressing slowly. Although a lot of reports on new antifungal drugs, only three new antifungal drugs (Isavuconazole, ibrexafungerp, and rezafungin) and two new formulations of posaconazole were marketed over the last decade. Chinese botanical medicine has advantages in the treatment of drug-resistant VVC, such as outstanding curative effects and low adverse reactions, which can improve patients’ comfort and adherence to therapy. Kangbainian lotion (KBN), a Chinese botanical formulation, has achieved very good clinical effects in the treatment of VVC. In this study, we investigated the antifungal and anti-inflammatory effects of KBN at different doses in fluconazole-resistant (FLC-resistant) VVC model mice. We further studied the antifungal mechanism of KBN against FLC-resistant *Candida albicans* (*C. albicans*) and the anti-inflammatory mechanism correlated with the Dectin-1 signaling pathway. *In vivo* and *in vitro* results showed that KBN had strong antifungal and anti-inflammatory effects in FLC-resistant VVC, such as inhibiting the growth of *C. albicans* and vaginal inflammation. Further studies showed that KBN inhibited the biofilm and hypha formation, reduced adhesion, inhibited ergosterol synthesis and the expression of ergosterol synthesis-related genes *ERG11*, and reduced the expression of drug-resistant efflux pump genes *MDR1* and *CDR2* of FLC-resistant *C. albicans in vitro*. In addition, *in vivo* results showed that KBN reduced the expression of inflammatory factor proteins TNF-α, IL-1β, and IL-6 in vaginal tissues, and inhibited the expression of proteins related to the Dectin-1 signaling pathway. In conclusion, our study revealed that KBN could ameliorate vaginal inflammation in VVC mice caused by FLC-resistance *C. albicans*. This effect may be related to inhibiting the growth of FLC-resistance *C. albicans* and Dectin-1 signaling pathway activation.

## Introduction

Vulvovaginal candidiasis (VVC) is a common gynecological disease in clinical, and surveys reported that 70–75% of women will suffer from VVC at least once during their lifetime (Farr et al., 2021). Even worse, up to 140 million of these women will develop recurrent VVC (RVVC) defined as ≥4 cases within 1 year (Mckloud et al., 2021). VVC is characterized by redness, burning, and itching of the vulva and vaginal mucosa, frequently accompanied by thick white vaginal secretions (Peters et al., 2014). It even causes dysuria and dyspareunia (Faria et al., 2017), which has a great impact on the quality of life of women in general.

*Candida albicans* (*C. albicans*) is the most common species identified in patients with VVC (76–89%), followed by *C. glabrata*, *C. tropicalis*, *C. parapsilosis*, etc (Achkar and Fries, 2010). A variety of azoles are available for the treatment of VVC caused by *C. albicans* both as topical and systemic agents (Sobel and Sobel, 2018). However, antifungal drugs can have adverse effects such as headaches and gastrointestinal disorders (Felix et al., 2019). Currently, the resistance to available antifungal drugs is a growing problem. In general, widespread use of azoles has significantly increased azoles exposure and resulted in acquired azoles resistance (Pristov and Ghannoum, 2019). Even in drug-naive patients, an increase in resistant *Candida* species has been observed (Arendrup, 2014). Therefore, there is an urgent need to develop new antifungals. However, research on new antifungal drugs is progressing slowly. Although a lot of reports on new antifungal drugs, only three new antifungal drugs (Isavuconazole, ibrexafungerp, and rezafungin) and two new formulations of posaconazole were marketed over the last decade (Van Daele et al., 2019).

The resistance mechanism of *C. albicans* has the characteristics of multi-factor and multi-level regulation, mainly concentrated in the following three aspects: mutation or overexpression of *ERG11* gene related to ergosterol synthesis (Flowers et al., 2015; Teymuri et al., 2015), increased expression of genes encoding drug efflux pumps (such as *MDR1*, *CDR1*, *CDR2*) (Mukherjee et al., 2003), and biofilm formation (Van Acker et al., 2014). Overexpression of *ERG11* increases the amount of *Erg11p* enzyme and results in elevating ergosterol synthesis, which leads to resistance to the azole drugs (Alizadeh et al., 2017). *Erg11p* is a target enzyme of azole drugs, and the high concentrations of target enzyme create the need for higher intracellular fluconazole (FLC) concentrations to inhibit all of the enzyme molecules in the cell (Pfaller et al., 2006). The multidrug resistance phenotype in *C. albicans* has previously been demonstrated to be linked to proteins encoded by *MDR1*, *CDR1*, and *CDR2* genes, which pump drugs from the fungal cells (Sanglard et al., 1996; Mukherjee et al., 2003).

A large amount of evidence indicates that Dectin-1 regarded as a pattern recognition receptor (PRR) and plays an important role in defense against fungal infection (Drummond and Brown, 2011). Dectin-1, a transmembrane protein, is a member of the C-type lectin receptor (CLR) family. The Dectin-1 receptor is served as the PRR for *β*-glucan in the fungal cell wall, and it triggers a signaling cascade that can activate NF-κB, which leads to the production of TNF-α、IL-1β、IL-6 (Falsetta et al., 2015; Yang et al., 2018; Tam et al., 2019).

Chinese botanical medicine has advantages in the treatment of drug-resistant VVC, such as outstanding curative effects and low adverse reactions, which can improve patients’ comfort and adherence to therapy. According to traditional Chinese medicine (TCM) theories, excess damp-heat is considered to be the key pathological factor in the pathogenesis of VVC (Yang et al., 2018). Kangbainian lotion (KBN) has been clinically used for VVC treatment for years with the effects of clearing heat and removing dampness (Huang et al., 2019). KBN is composed of *Coptis chinensis* Franch (Huanglian), *Saururus chinensis* (Lour.) Baill (Sanbaicao), *Isatidis folium* (Daqingye), *Celosia cristata* L. (Amaranthaceae) (Jiguanhua), *Elsholtzia ciliata* (Thunb.) Hyland (Xiangru), *Sophora flavescens* Alt. (Kushen), *Stemona tuberosa* Lour. (Baibu), *Gentiana scabra* Bunge (Longdan), *Eugenia caryophyllata* Thunb. (Dingxiang), *Cinnamomum camphora* (L.) J.Presl (Borneol, Bingpian). These botanical drugs or extracts are considered potential candidates for treating VVC. Their bioactive properties include anti-fungal (Liu et al., 2012; Yang et al., 2015; Niu et al., 2019) and anti-inflammatory (Lee et al., 2014; Yang et al., 2019; Ji et al., 2020; Pudziuvelyte et al., 2020). The previous research found that KBN significantly alleviated the vaginal inflammation in VVC model mice. However, the mechanism of KBN in the treatment of fluconazole-resistant (FLC-resistant) VVC needs further study.

In this study, we investigated the antifungal and anti-inflammatory effects of KBN at different doses in FLC-resistant VVC model mice. We further studied the antifungal mechanism of KBN against FLC-resistant *C. albicans* and the anti-inflammatory mechanism correlated with the Dectin-1 signaling pathway. Our findings provide better insight into the molecular mechanism of KBN as a treatment for VVC.

## Materials and Methods

### Preparation and HPLC Analysis of KBN

#### Preparation of KBN

The composition of the Chinese botanical formulation KBN, plant parts used, and botanical drug dose are presented in table 1. The total dosage of KBN is 206 g. Borneol was purchased from Yunnan Linyuan Spice Co., Ltd. (Yunnan, China), the rest of the botanical drugs were purchased from Guangzhou Zhixin Chinese Medicine Pieces Co., Ltd. (Guangzhou, China). Except for borneol, the rest of the botanical drugs were extracted in a 6-and 4-fold volume of 80% ethanol at 100°C for 1.0 h. After filtrating, both filtrates were mixed and concentrated to 1 g/mL raw botanical drugs. Then the concentrated decoction was added 1 g of borneol dissolved in little ethanol to obtain KBN for *in vitro* experiments. To obtain 0.4, 0.2, and 0.1 g/mL KBN preparation containing 4% Avicel (Dupont, USA) for *in vivo* experiments, 200, 100, and 50 mL of KBN were respectively added 20 g of Avicel CL-611with stirring (12,000 r/min) and ultrapure water to 500 mL.

**TABLE 1:** The botanical composition of KBN.

Scientific name	Pinyin name	Plant part used	Botanical drug dose (g)	Composition (%)
*Coptis chinensis* Franch	Huanglian	Rhizome	75.0	36.41
*Saururus chinensis* (Lour.) Baill	Sanbaicao	Rhizome	37.5	18.20
*Isatidis folium*	Daqingye	Leaf	20.0	9.71
*Celosia cristata* L. (Amaranthaceae)	Jiguanhua	Flower	20.0	9.71
*Elsholtzia ciliata* (Thunb.) Hyland	Xiangru	Whole grass	12.5	6.07
*Sophora flavescens* Alt	Kushen	Root	12.5	6.07
*Stemona tuberosa* Lour	Baibu	Root	12.5	6.07
*Gentiana scabra* Bunge	Longdan	Rhizome	10.0	4.85
*Eugenia caryophyllata* Thunb	Dingxiang	Flower bud	5.0	2.43
*Cinnamomum camphora* (L.) J.Presl	Bingpian	Branch and leaf extraction	1.0	0.48

#### Component Analysis of KBN by HPLC

**FIGURE 1:**
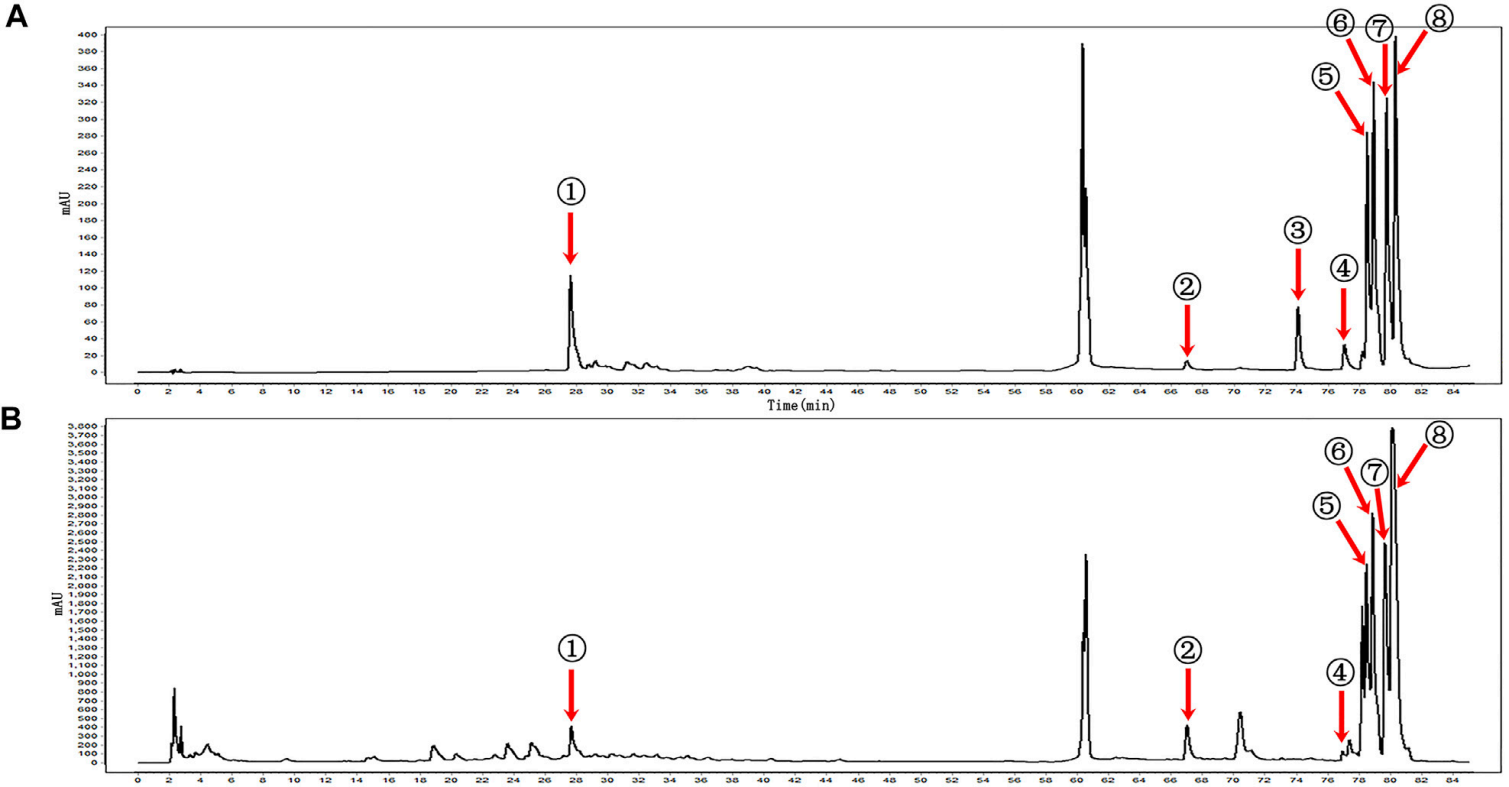
HPLC profiles of the main components of KBN. **(A)** Mixed reference; **(B)** KBN; ① Gentiopicrin; ② Eugenol; ③ Carvesol; ④ Indirubin; ⑤ Epiberberine; ⑥ Coptisine; ⑦ Palmatine; ⑧ Berberine.

KBN was determined by the Agilent 1260 high-performance liquid chromatography (HPLC) system (Agilent Technologies, United States) equipped with a VWD detector and a Kromasil 100-5-C18 column (250 × 4.6 mm, 5 μm). The mobile phase consisted of A and B with a gradient elution as follows: 0–10 min for 2–5% B, 10–20 min for 5–10% B, 20–25 min for 10–15% B, 25–55 min for 15% B, 55–60 min for 15–30% B, 65–70 min for 40–50% B, 70–85 min for 50–70% B. A was composed of 0.057 mol/L of sodium dihydrogen phosphate and 0.014 mol/L of sodium dodecyl sulfate and then adjusted pH to 4 with phosphoric acid. B was acetonitrile. The detection wavelength was set at 230 nm, the column temperature was maintained at 40°C, the loading volume was 5 μL and the flow rate was 1.0 mL/min. According to the retention time and peak area of the reference standard, a total of 7 components of KBN was detected, namely, gentiopicrin, eugenol, indirubin, epiberberine, coptisine, palmatine, and berberine (Figure 1).

### *C. albicans* Strains and Culture

Four *C. albicans* strains were tested in this study. The quality control sensitive strain CMSS(F) 98001 was provided by the China National Institute for the Control of Pharmaceutical and Biological Products. The clinically FLC-resistant *C. albicans* isolates (901, 904, and 311) were donated by Dr. Jiang from the Second Military Medical University. Two distinct colonies of approximately 1 mm diameter were picked and inoculated in 1 mL of yeast extract-peptone-dextrose (YPD) medium (1% yeast extract, 2% peptone, 2% dextrose) at 35°C with constant shaking (200 rpm) for 16 h. Then, the cells were harvested by centrifugation, re-suspended in Roswell Park Memorial Institute (RPMI) 1640 medium with 0.165 M 3-(N-Morpholino) propane sulfonic acid (MOPS) (pH7.0), and counted after serial dilution by a hemocytometer.

### Antifungal Susceptibility Test and Time-Kill Curve Analysis of KBN on *C. albicans*

#### Antifungal Susceptibility Test of KBN on *C. albicans*

Using a broth microdilution protocol of the Clinical and Laboratory Standards Institute (CLSI) M27-A3 method, an antifungal susceptibility test was carried out in 96-well tissue culture plates (Corning, USA) with a few modifications (Yan et al., 2019). In brief, the initial concentration of *C. albicans* in the RPMI 1640 medium was 2.0 × 10^3^ colony-forming units (CFU)/mL, the final concentration ranged from 0.039 to 20 mg/mL for KBN, 0.125–64 μg/mL for FLC, and 0.015–8 μg/mL for ketoconazole. To further test the minimal inhibitory concentration (MIC) of FLC-resistant *C. albicans*, the final concentration ranged from 4 to 2,048 μg/mL for FLC. Plates were incubated at 35°C for 48 h. MIC endpoints were read as previously described (Li et al., 2019; Yan et al., 2019; Yong et al., 2020). In brief, the MIC was determined as the lowest concentration of the drug that inhibited planktonic *C. albicans* growth by 80% compared with that of the drug-free *C. albicans*. The MIC of fluconazole against *C. albicans* was also tested with the same criteria. Experiments were performed in triplicate.

#### Time-Kill Curve Assay of KBN on *C. albicans*

To further study the antifungal activity of KBN, a time-kill curve assay was performed as described previously (Yong et al., 2020). Time-kill experiments were conducted in RPMI 1640 medium with MOPS. The final concentrations were 12.5, 6.25 and 3.125 μg/mL for KBN, 2 μg/mL for FLC, and 4.0 × 10^4^ CFU/mL for *C. albicans*. A drug-free group served as the negative control. Cultures were incubated at 35°C with constant shaking (200 rpm) after different treatments. 50 μL of aliquots were obtained from each suspension at 0, 3, 6, 12, 24, 36, and 48 h, and serially diluted in sterile phosphate balanced solution (PBS). 50 μL of the diluted fungal suspension was plated on sabouraud dextrose agar (SDA) plates, and colonies were counted following incubation at 35°C for 36 h.

### Effect of KBN on the VVC Model Mice Caused by FLC-Resistant *C. albicans* 901

As previously described (Yano and Fidel, 2011), the VVC model mice were used to demonstrate the antifungal effect of KBN *in vivo*. Briefly, 6 days before inoculation, SPF KM female mice (Guangdong Medical Laboratory Animal Center) were injected with 0.1 mL of *β*-estradiol (0.2 mg/mL, Hangzhou Animal Medicine Factory, China) subcutaneously in the lower abdominal. Estrogens were injected every other day, three times in 6 days. After estradiol treatments, infection of the vagina was performed by inoculating the mice intravaginally with 20 μL of FLC-resistant *C. albicans* 901 suspensions with a concentration of 1.0 × 10^8^ CFU/mL every day, seven times a week. After the establishment of VVC model mice, KBN (0.4, 0.2, and 0.1 g/mL) and FLC (2 mg/mL) were separately delivered into the vagina once a day for 3 weeks, 30 μL per day. Drug-free groups were used as negative control and positive control. On day 1 before administration, day 3, 7, and 14 after treatment, 100 μL of vaginal lavage was collected and cultured on the SDA plates for colony counts. The CFU was counted to analyze the infection burdens. 10 μL of lavage fluid was observed by Gram staining. All mice were killed with anesthetics and the vaginal tissues were observed by hematoxylin and eosin (H&E) staining, periodic acid schiff (PAS) staining, and scanning electron microscopy (SEM). The study was approved by the Animal Ethics Committee of the Institute of Science and Technology at Guangzhou University of Chinese Medicine (Guangzhou, China).

### The Mechanism of KBN Inhibiting FLC-Resistant *C. albicans*

#### The Effect of KBN on the Biofilm Formation by XTT Reduction Assay

The 2,3-bis-(2-methoxy-4-nitro-5-sulfophenyl)-2H-tetrazolium-5-carboxanilide (XTT) reduction assay was conducted as described previously (Ramage et al., 2001) with slight modifications. Briefly, 100 µL of *C. albicans* with an initial concentration of 1.0 × 10^6^ CFU/mL was cultured in a sterile 96-well plate at 37°C for 1.5 h and discarded. Biofilm cells were washed with PBS, cultured in 1640 medium with or without drugs at 37°C for 24 h, and discarded. Biofilm cells were washed with PBS and then incubated in 200 μL PBS with 0.5 mg/mL of XTT and 1 µM of menadione at 37°C for 1 h. 100 μL of supernatant was transferred to new microtiter plates, and optical densities were measured at 492 nm.

#### The Effect of KBN on the Hyphae Growth

The hypha-inducing media (RPMI 1640 medium with MOPS) was used to test the effect of KBN on the hyphal formation. *C. albicans* cells (1.0 × 10^5^ CFU/mL) were suspended in the hypha-inducing media containing 12.5, 6.25, and 3.125 mg/mL of KBN and added to 12-well tissue culture plates. The plates were incubated at 37°C for 3 h. The morphology of *C. albicans* was observed and photographed under an inverted microscope.

#### The Effect of KBN on the Adhesion to VK2/E6E7

VK2/E6E7 kindly gifted from Dr. Chen (Tongji Medical College) is a vaginal epithelial cell. The cells were maintained and passaged in CnT-Prime epithelial culture medium (CELLnTEC, Switzerland) at 37°C with 5% CO_2_. The adhesion test was conducted as described previously (Gao et al., 2019), with slight modifications. 1.0 × 10^5^ cells/well of *C. albicans* were inoculated into VK2/E6E7 monolayer cultures in a 24-well plate with or without KBN at 37°Cwith 5% CO_2_ for 1.5 h. All the mixtures of cell monolayer and fungal cells were fixed with 4% paraformaldehyde for 15 min, stained with 30 μg/mL CFW (Sigma-Aldrich, United States) for 15 min, and then photographed under a fluorescent microscope.

#### The Effect of KBN on the Ergosterol by HPLC

Total sterols were extracted from whole cells according to a previous report with slight modifications (Xu et al., 2017). Briefly, *C. albicans* with an initial concentration of 2.0 × 10^6^ CFU/mL were cultured in RPMI 1640 medium with or without KBN at 35°C for 48 h. 6.0 mL of each sample were collected, added 15 mL of saponifier (methanol solution with 10% KOH), and then incubated in an 80°C water bath for 90 min. The total sterols were extracted thrice with 15 mL of petroleum ether (boiling range 30°C-60°C) and incubated in a 60°C water bath until petroleum ether layer volatilized completely. The resulting samples were dissolved in 6 mL of methanol, filtered with a 0.45 μm microporous membrane, and used for HPLC quantification. The ergosterol standard (0.125–64 μg/mL) dissolved in methanol and the sample-derivatized sterols were determined by the Agilent 1260 HPLC system equipped with a VWD detector and a Kromasil 100-5-C18 column (250 × 4.6 mm, 5 μm). The mobile phase consisted of 95% methanol and 5% water. The detection wavelength was set at 282 nm, the column temperature was maintained at 30°C, the flow rate was 1.0 mL/min. The ergosterol standard loading volume was 10 μL, and the sample-derivatized sterols loading volume was 100 μL.

#### The Effect of KBN on the Gene Expression by RT-PCR

Reverse transcription-polymerase chain reaction (RT-PCR) was conducted as described previously (Zhong et al., 2017) with slight modifications. Briefly, *C. albicans* cells with an initial concentration of 3.0 × 10^6^ CFU/mL were cultured in YPD medium at 35°C for 6 h. *C. albicans* cells were collected and washed, and total RNA was isolated by an EASYspin yeast RNA rapid extraction kit (RN10, Aidlab Biotechnologies, China). cDNA was obtained by a reverse transcription reaction performed with a reverse transcription kit (AG11728, Accurate Biotechnology, China). RT-PCR was performed with a 7500 real-time PCR system (Thermo Fisher, United States), and specific primers were synthesized by Sangon Biotech (Table 2). SYBR green (AG11718, Accurate Biotechnology, China) was used to monitor the amplified products. The expression of each gene was normalized to that of *18S rRNA*. The relative genes expression were quantified by the 2^−△△Ct^ method and triplicate independent experiments were performed to obtain a mean value.

**TABLE 2:** Primer sequences used in the RT-PCR analysis.

Targeted genes		Gene sequence (5′ to 3′)
*18s rRNA*	Forward primer	AAT​TAC​CCA​ATC​CCG​ACA​C
	Reverse primer	TGC​AAC​AAC​TTT​AAT​ATA​CGC
*ERG11*	Forward primer	TTGGTGGTGGTAGACATA
	Reverse primer	TCTGCTGGTTCAGTAGGT
*MDR1*	Forward primer	TTT​GGT​TCA​TGT​GTA​TCA​TTT​CTG​G
	Reverse primer	GAA​TAT​AAA​TAA​AGG​CAG​CAA​TGA​C
*CDR1*	Forward primer	GGT​CAA​CTT​GTA​ATG​GGT​C
	Reverse primer	AGGACGATAAAGGGCATA
*CDR2*	Forward primer	GCCAATGCTGAACCGACA
	Reverse primer	ACCAGCCAATACCCCACA

### Anti-Inflammatory Mechanism of KBN on VVC Model Mice Caused by FLC-Resistant *C. albicans* 901 by Western Blot

The vaginal tissues were lysed and homogenized in RIPA buffer with phenylmethylsulfonyl fluoride (PMSF) and quantified with a BCA kit (Keygen Biotech, KGP902, China). The protein samples (30 μg/lane) were separated using SDS-PAGE (Beyotim, P0012A, China) and transferred to polyvinylidene difluoride membranes (PVDF) (Merck, IPVH00010, United States) after electrophoresis. The membranes were blocked with 5% skimmed milk for 3 h, followed by incubation with TNF-α (1:1,000, Abcam, ab205587, United States), IL-1β (1:1,000, Abcam, ab234437, United States), IL-6 (1:1,000, Abcam, ab229381, United States), Dectin-1 (1:1,000, Abcam, ab140039, United States), Syk (1:1,000, Cell Signaling Technology, 13198T, United States), PLCγ-2 (1:1,000, Cell Signaling Technology, 3872T, United States), CARD9 (1:500, Affinity, DF8387, China), NF-κB (1:1,000, Cell Signaling Technology, 8242T, USA) and *β*-actin (1:2,000, Affinity, AF7018, China) antibodies overnight at 4°C. Then, the membranes were incubated with an HRP-conjugated antibody (1:5,000, Affinity, S0001, China) at room temperature for 1 h. The membranes were visualized with a chemiluminescence (ECL) kit by a Tanon 5200 system (Shanghai, China). ImageJ software (Version 1.52a) was used to measure the protein bands based on that of *β*-actin.

### Statistical Analysis

SPSS 20.0 software was used for statistical analysis. The results were expressed as the mean ± standard deviation (SD). One-way ANOVA was utilized for more than two groups. The Levene was used to test the homogeneity of variance, the least significant difference test (LSD-t) was used for inter-group comparisons when the variance was homogeneous, and Dunnett’s T3 was used for inter-group comparisons when the variance was uneven. *p* < 0.05 was recognized as statistically significant.

## Results

### KBN Inhibits *C. albicans* Growth

**FIGURE 2:**
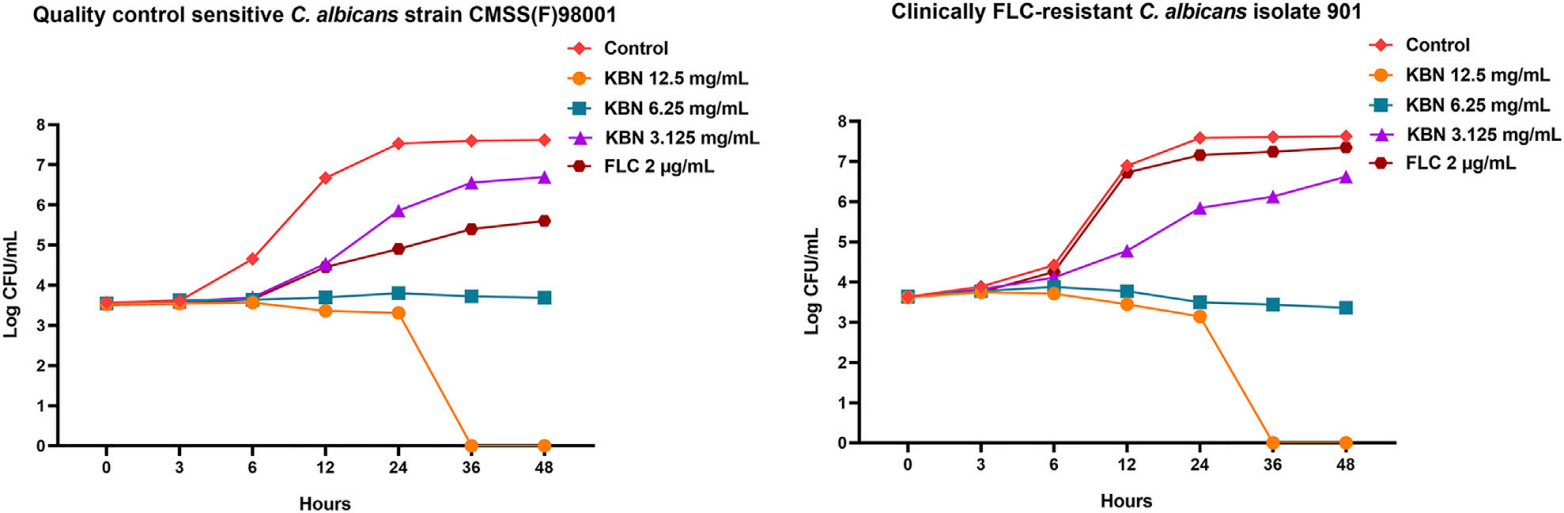
Effects of KBN on the time-killing curve of *C. albicans*. The quality control sensitive *C. albicans* strain CMSS(F) 98001 and the clinically FLC-resistant *C. albicans* isolate 901 were used.

We first determined the MIC values of KBN on *C. albicans* strains, including the quality control sensitive CMSS(F) 98001 and the clinically FLC-resistant isolates (901, 904, and 311). The MIC of KBN against planktonic *C. albicans* was 6.25 mg/mL, for all the tested strains (Table 3). The dynamic antifungal effect of the drug could be monitored by the time-kill curve assays for CMSS(F) 98001 and 901 strains (Yong et al., 2020). The results showed that KBN appeared to exhibit strong antifungal activity against *C. albicans*. Especially, 12.5 mg/mL of KBN resulted in 99.9% killing within 36 h against *C. albicans* (Figure 2). It should be noted that KBN exhibited similar antifungal activity on the FLC-sensitive strain and resistant strains.

**TABLE 3:** MIC of KBN against *C. albicans*.

*C. albicans* Strains	KBN	FLC	Ketoconazole
	mg/mL	μg/mL	μg/mL
98001	6.25	1	<0.015
901	6.25	1,024	>8
904	6.25	512	>8
311	6.25	512	>8

### KBN Inhibits the Proliferation of *C. albicans* and Vaginal Inflammation in VVC Model Mice Caused by FLC-Resistant *C. albicans*

**FIGURE 3:**
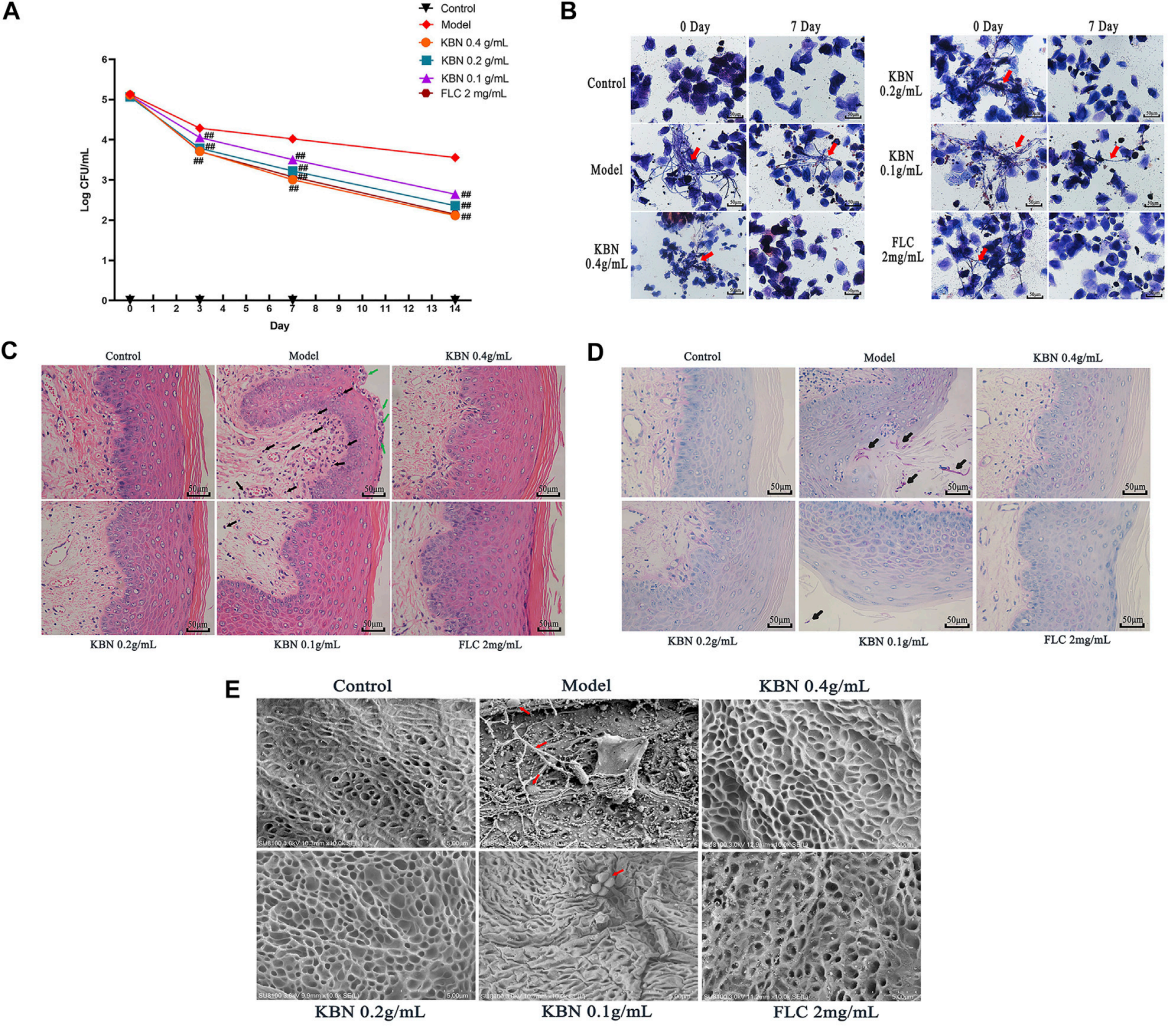
The vaginal fungal burden and the intensity of vaginal tissue inflammation in mice infected with FLC-resistant *C. albicans* 901 after the treatment of KBN. **(A)** Vaginal fungal burden analyses by colony counting in infected mice. Each value is presented as the mean ± SD, *N* = 10. ^##^ *p* < 0.01 as compared to the values from the model group. **(B)** The formation of hyphae in vaginal excretion after KBN treatment was indicated by Gram staining (400 ×). The red arrows represent visible hyphae in vaginal excretions. **(C)** Histopathological analysis of vaginal tissue in infected mice by H&E stain (400 ×). The green arrow shows microabscesses and the black arrow shows neutrophils, indicating the presence of inflammation. **(D)** Histopathological analysis of vaginal tissue in infected animals by PAS (400 ×). The black arrow shows *C. albicans*. **(E)** Morphology of the vaginal epithelium by SEM after infection and KBN treatment (10 K ×). The red arrows represent *C. albicans*.

VVC is mainly caused by *C. albicans* and infections rely on fungal morphogenesis and biofilm formation (Fazly et al., 2013). This mouse vaginal infection model is ideal for evaluating *in vivo* antifungal potency of KBN. We assessed the antifungal effects of KBN in vaginal infection by colony counts, Gram stain, H&E stain, PAS stain, and SEM (Figure 3). All three KBN groups exhibited markedly reduced fungal burden from day 3 onwards compared with the model group (*p* < 0.01), and KBN treatment reduced the fungal loads in a dose-dependent manner (Figure 3A). From the images of lavage fluid Gram staining, vaginal PAS staining, and scanning electron, we observed a lot of criss-cross filamentous cells in the model (Figures 3B,D,E). After H&E staining, we observed that the vaginal mucosa was seriously injured in the model, included cellular edema, inflammatory cell infiltration (Figure 3C). By the management of KBN, the cellular symptoms were relieved to different degrees. Noticeably, when exposed to 0.4 or 0.2 g/mL of KBN, the vaginal mucosa was largely recovered, without cell edema and inflammatory cell infiltration. Interestingly, KBN restrained the formation of mycelium *in vivo*, which was consistent with its hyphal inhibition activity *in vitro* (Figures 5, 6).

### The Mechanism of KBN Inhibiting FLC-Resistant *C. albicans* 901

#### KBN Inhibits the Biofilms Formation

**FIGURE 4:**
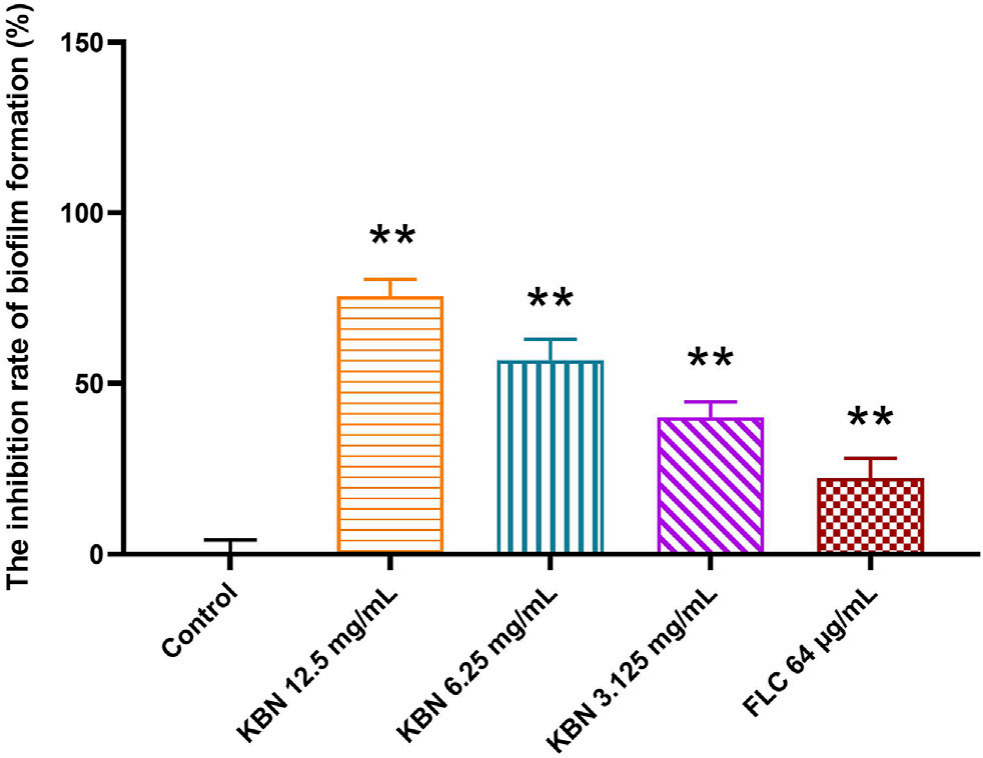
Effects of different concentrations of KBN on FLC-resistant *C. albicans* 901 biofilm formation. Data are expressed as the mean ± SD of the independent assays in sextuplicate. ^∗∗^ *p* < 0.05 as compared to the control group.

KBN significantly inhibited biofilm formation of FLC-resistant *C. albicans* 901 in a dose-dependent manner (Figure 4). More specifically, 3.125 mg/mL of KBN inhibited biofilm formation by 40.15%, and the inhibitory effect on biofilm formation was enhanced when the concentration of KBN was increased. 6.25 mg/mL of KBN inhibited biofilm formation by 56.86%, and 12.5 mg/mL of KBN inhibited biofilm formation by 75.54%. 64 μg/mL of FLC only inhibited biofilm formation by 22.23%.

#### KBN Inhibits the Hyphae Growth

**FIGURE 5:**
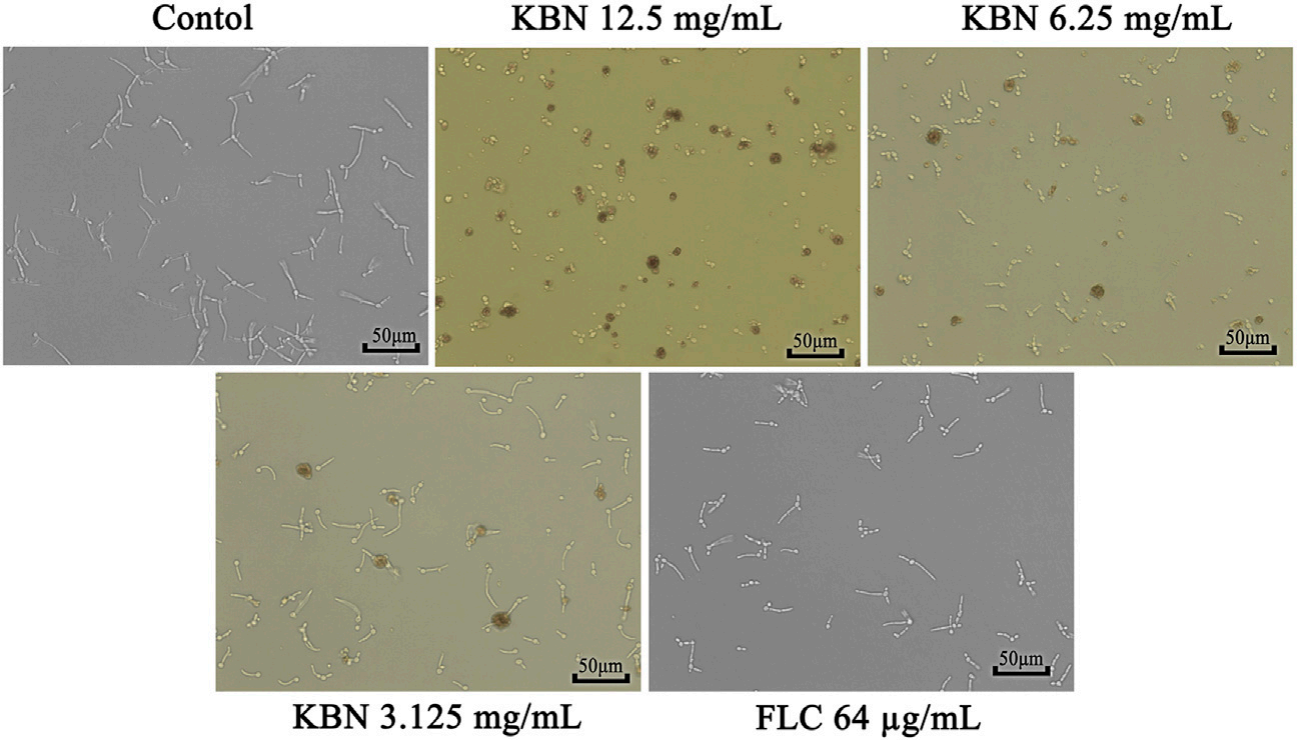
Effects of KBN on hyphae growth of FLC-resistant *C. albicans* 901. The cellular morphology was photographed after incubating at 37°C for 3 h.

Since hypha growth is a key factor in *C. albicans* biofilm formation and virulence, we tested the effect of KBN on hypha growth. In the absence of KBN, FLC-resistant *C. albicans* 901 underwent hyphal initiation, produced elongated and regular hyphal cells (Figure 5). When 12.5 or 6.25 mg/mL of KBN was added, the hypha growth was remarkably inhibited. Specifically, the hypha growth was completely inhibited by 12.5 mg/mL of KBN as only yeast cells were observed.

#### KBN Inhibits the Adhesion to VK2/E6E7

**FIGURE 6:**
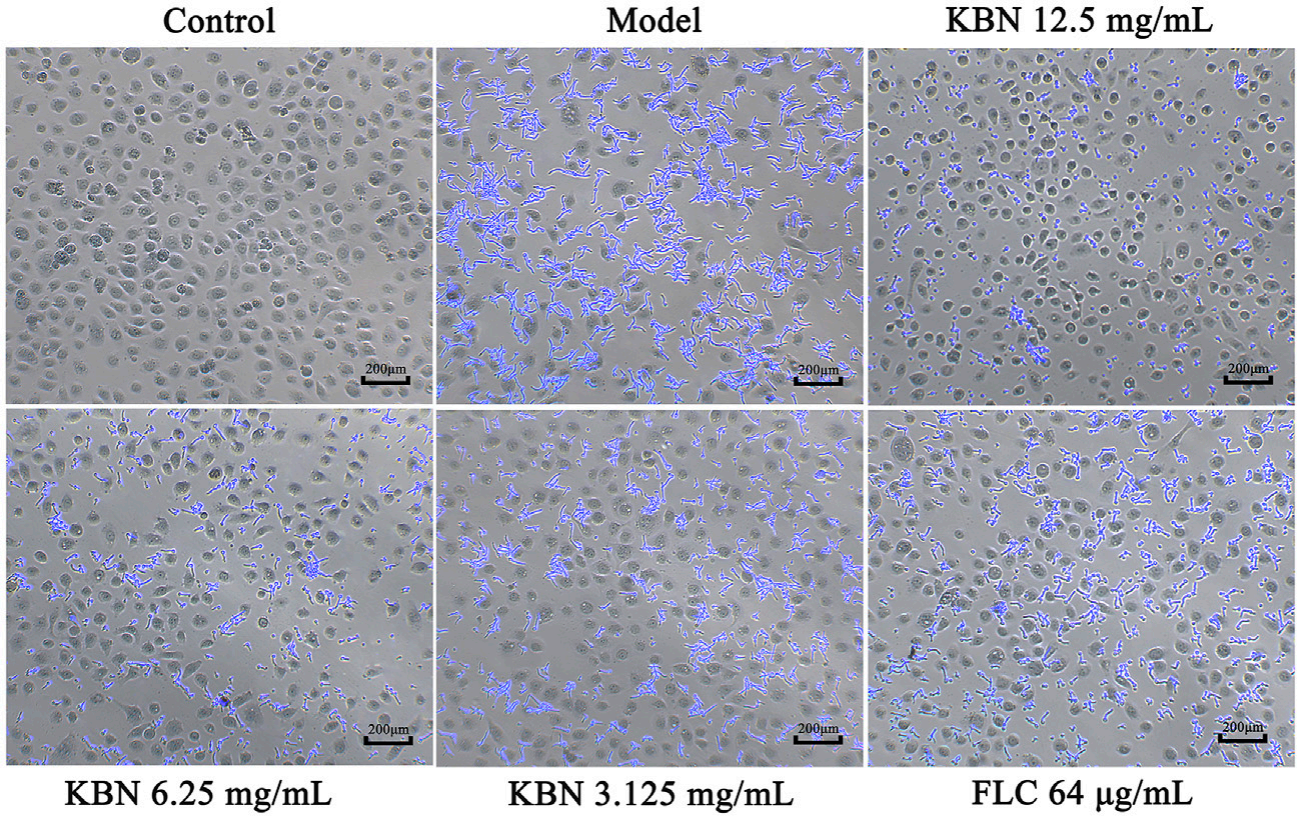
KBN inhibited the adhesion of FLC-resistant *C. albicans* 901 to VK2/E6E7 cells. The treatment concentration for KBN were 12.5, 6.25, and 3.125 mg/mL.

We further analyzed the effect of KBN on FLC-resistant *C. albicans* 901 interacting with VK2/E6E7 by analyzing adhesion. 12.5 or 6.25 mg/mL of KBN significantly inhibited the adhesion of FLC-resistant *C. albicans* 901 to the surface of VK2/E6E7 cells and reduced the damage of *C. albicans* to VK2/E6E7 cells (Figure 6). In addition, 12.5 or 6.25 mg/mL of KBN significantly inhibited the hyphae growth of FLC-resistant *C. albicans* 901.

#### KBN Reduces the Content of Ergosterol and Overexpression of Related Gene *ERG11*

**FIGURE 7:**
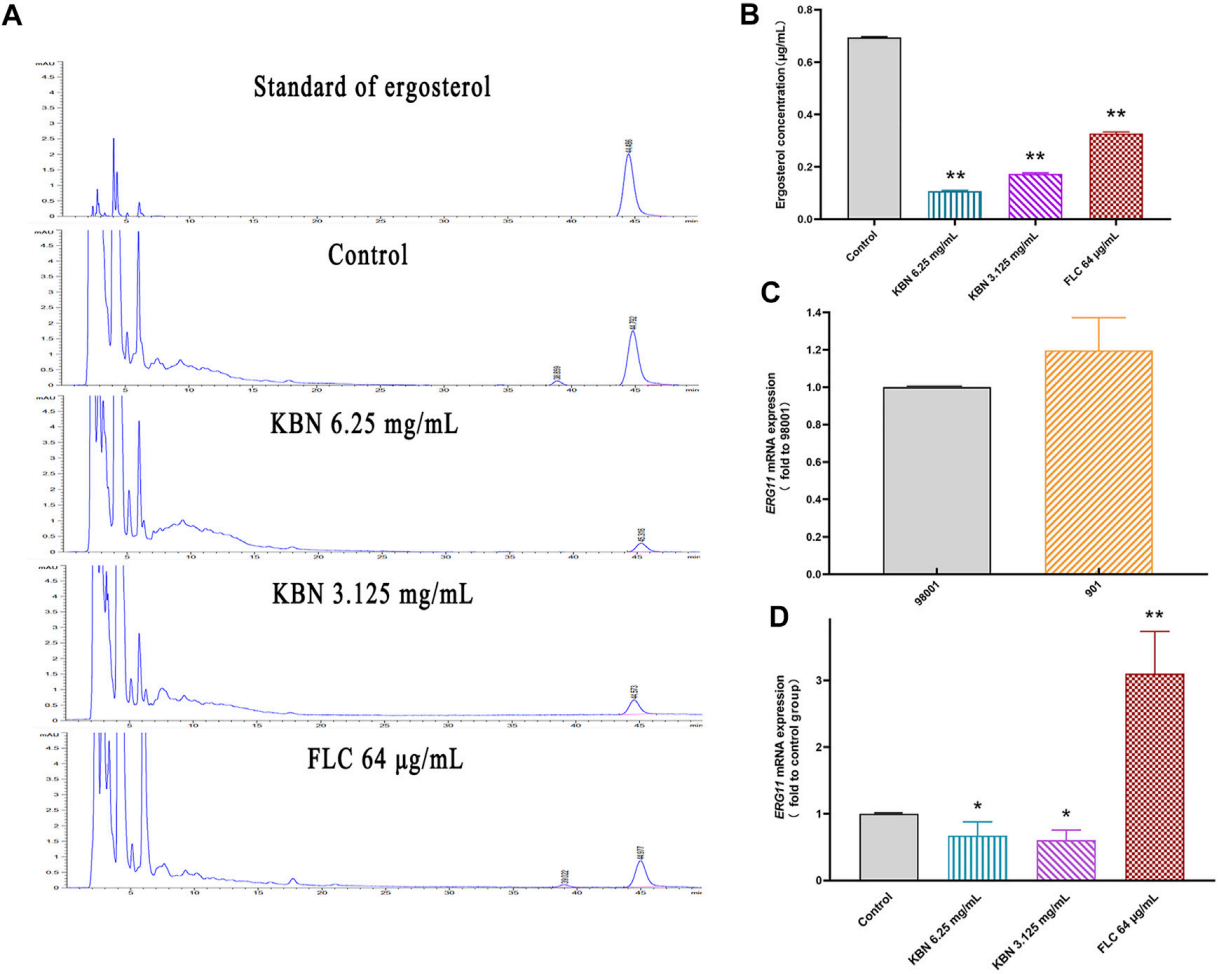
Effect of KBN on ergosterol content of FLC-resistant *C. albicans* 901 and expression of related gene *ERG11*. **(A)** Quantitative analysis of cellular ergosterol in FLC-resistant *C. albicans* 901 treated with KBN or FLC for 48 h by HPLC. **(B)** Ergosterol content of cells treated with KBN. Values were described as ergosterol (μg/mL). **(C)** The expression of ergosterol synthesis gene *ERG11* in FLC-resistant *C. albicans* 901 and sensitive *C. albicans* 98001. **(D)** Changes in the expression of ergosterol synthesis gene *ERG11* in FLC-resistant *C. albicans* 901 treated with KBN or FLC for 6 h. The gene was examined by RT-PCR with gene-specific primers. All values are expressed as the mean ± SD of triplicate assays. ^*^ *p* < 0.05 versus control group, ^**^ *p* < 0.01 versus control group.

In this study, we investigated the resistance mechanism of ergosterol in FLC-resistant *C. albicans* 901 and the effect of KBN on it. First, the cellular ergosterol levels in FLC-resistant *C. albicans* 901 were measured after KBN or FLC treatment by HPLC analysis. The ergosterol content significantly treated with 6.25 or 3.125 mg/mL of KBN decreased in the cells compared with the control group (*p* < 0.01) (Figures 7A,B). Changes in the expression of ergosterol synthesis-related gene *ERG11* in FLC-resistant *C. albicans* 901 and sensitive *C. albicans* 98001 were investigated by RT-PCR analysis. The expression of the *ERG11* gene in FLC-resistant *C. albicans* 901 was 1.2 times that of sensitive *C. albicans* 98001 (Figure 7C). The effect of KBN on the overexpression of *ERG11* in *C. albicans* 901 was further investigated. 6.25 or 3.125 mg/mL of KBN significantly reduced the expression level of *ERG11* (*p* < 0.05) (Figure 7D). Interestingly, FLC significantly increased the expression of *ERG11*.

#### KBN Reduces the Overexpression Levels of Efflux Pump Gene

**FIGURE 8:**
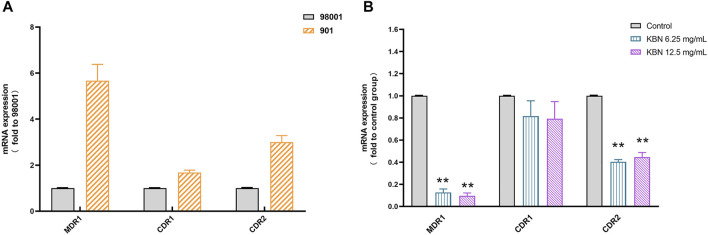
Relative expression of the efflux-pump genes in *C. albicans* strains. **(A)** The expression levels of efflux pump genes *MDR1*, *CDR1*, and *CDR2* in sensitive *C. albicans* 98001 and FLC-resistant *C. albicans* 901. **(B)** The expression levels of the efflux-pump genes *MDR1*, *CDR1*, and *CDR2* in FLC-resistant *C. albicans* 901 cells that were treated with KBN relative to those in untreated cells. Values represent the mean ± SD for three independent experiments. ^∗∗^ *p* < 0.01 versus control group.

First, the sensitive *C. albicans* 98001 was used as the quality control fungus to compare the expression of different efflux pump genes in FLC-resistant *C. albicans* 901. *18S rRNA* was used as an internal reference for each gene. There were no primer-dimers or nonspecific amplification products according to the melting curves and melting peaks of all the tested genes. The expression of *MDR1*, *CDR1*, and *CDR2* genes in FLC-resistant *C. albicans* 901 was higher than that of sensitive *C. albicans* 98001, which were 5.67, 1.68, and 3.00 times of those in FLC-sensitive *C. albicans* 98001, respectively (Figure 8A). For FLC-resistant *C. albicans* 901 strain, the *MDR1* and *CDR2* expression levels among treatments with 6.25 and 12.5 mg/mL of KBN significantly decreased (*p* < 0.01). However, there was no significant difference in the *CDR1* expression level among treatments (*p* > 0.05) (Figure 8B).

### Anti-Inflammatory Mechanism of KBN on VVC Model Mice Caused by FLC-Resistant *C. albicans* 901

#### KBN Decreased Inflammation Levels of Vaginal Tissue in VVC Model Mice

**FIGURE 9:**
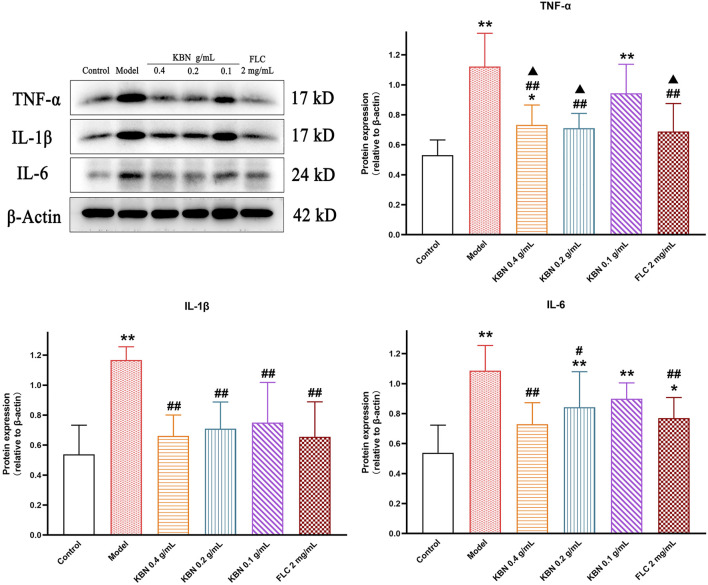
Effect of KBN on the protein expression of TNF-α, IL-1β, and IL-6 in the vaginal tissues. *N* = 6. Each value is presented as the mean ± SD. ^*^ *p* < 0.05, ^**^ *p* < 0.01 versus the control group; ^#^ *p* < 0.05, ^##^ *p* < 0.01 versus the model group; ^▲^ *p* < 0.05 versus the KBN 0.1 g/mL group.

To assess the level of inflammation induced by infection, vaginal tissues were collected and the protein levels of TNF-α, IL-1β, and IL-6 were analyzed by Western Blot. The protein expression levels of TNF-α、IL-1β, and IL-6 were significantly upregulated in the model group compared with that in the control group (*p* < 0.05), the expression of these proteins was significantly downregulated while KBN and FLC treatments (*p* < 0.05) (Figure 9).

#### KBN Regulated the Expression of Proteins Related to the Dectin-1 Signaling Pathway

**FIGURE 10:**
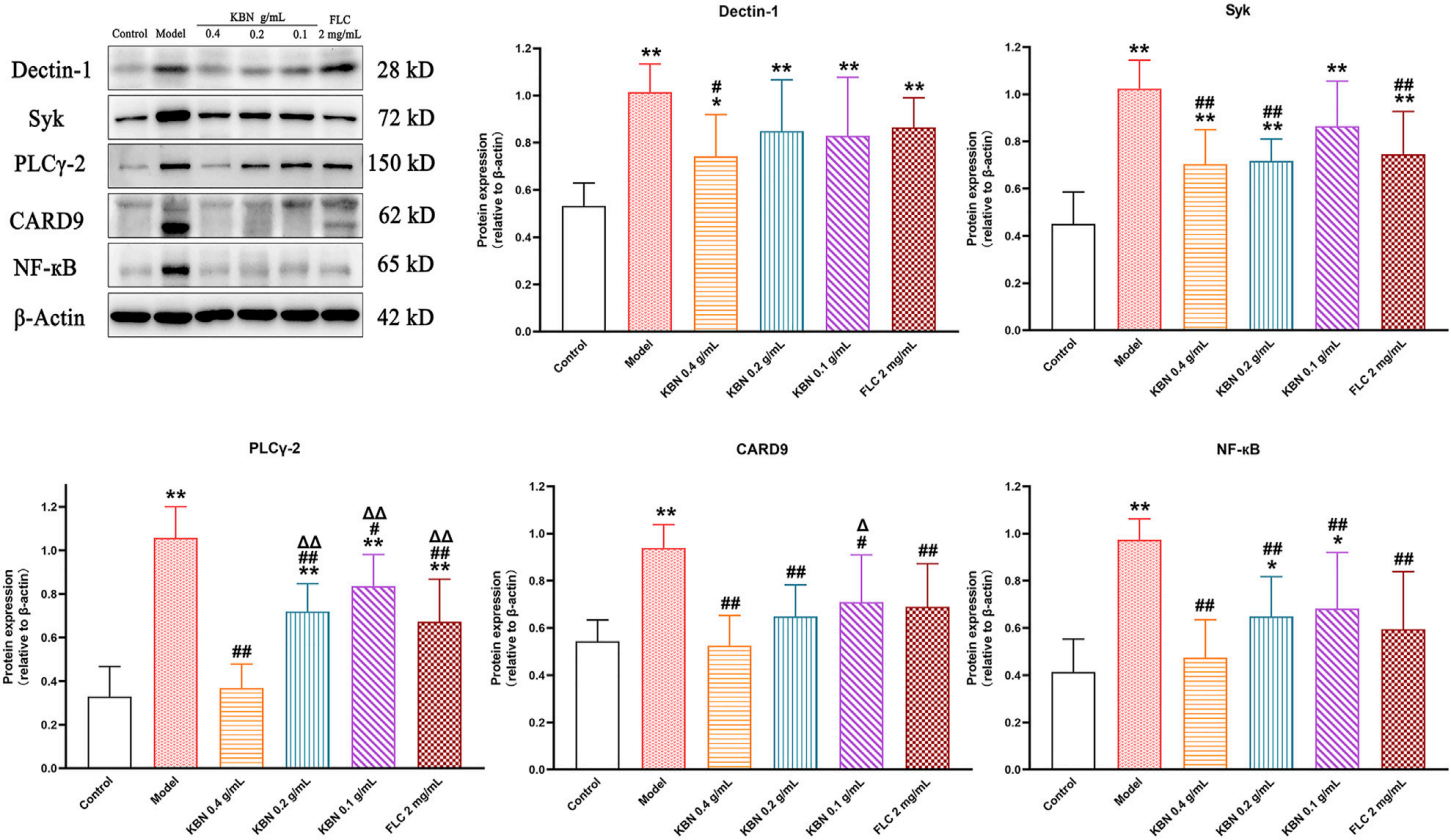
Effect of KBN on the protein expression of Dectin-1, Syk, PLCγ-2, CARD9, and NF-κB in the vaginal tissues. *N* = 6. Each value is presented as the mean ± SD. ^*^ *p* < 0.05, ^**^ *p* < 0.01 as compared to the control group; ^#^ *p* < 0.05, ^##^ *p* < 0.01 as compared to the model group; ^△^ *p* < 0.05, ^△△^ *p* < 0.01 as compared to the KBN 0.4 g/mL group.

To determine the potential molecular mechanism for the antifungal and anti-inflammatory effects of KBN, we studied the protein expression levels related to the Dectin-1 signaling pathway in vaginal tissues. The protein expression levels of Dectin-1, Syk, PLCγ-2, CARD9, and NF-κB showed a significant reduction in the model group (*p* < 0.05), and this trend was reversed after KBN treatment (*p* < 0.05) (Figure 10).

## Discussion

VVC is the second most common inflammatory disease in the female vagina, which has a serious impact on their quality of life (Dovnik et al., 2015). At present, an important factor in the refractory treatment of VVC is that fungus-inducing VVC has drug-resistance. Thus, there is an urgent need for new antifungal drugs that can efficiently treat VVC caused by drug-resistant *C. albicans*. In this study, we found that KBN had strong antifungal and anti-inflammatory effects in FLC-resistant VVC, such as inhibiting the growth of *C. albicans* and vaginal inflammation. Further studies showed that KBN inhibited the biofilm and hypha formation, reduced adhesion, inhibited ergosterol synthesis and the expression of ergosterol synthesis-related genes *ERG11*, and reduced the expression of drug-resistant efflux pump genes *MDR1* and *CDR2* of FLC-resistant *C. albicans in vitro*. In addition, *in vivo* results showed that KBN reduced the expression of the proteins of inflammatory factors TNF-α, IL-1β, and IL-6 in vaginal tissues, and inhibited the expression of the proteins related to the Dectin-1 signaling pathway.

Borneol, including natural borneol (D-borneol) and synthetic borneol (Borneol, isoborneol, and camphor) (Chinese Pharmacopoeia Commission, 2020), has been used in TCM clinical applications for more than 2,000 years (Wang et al., 2017). Our previous study showed that the MIC of borneol against sensitive or FLC-resistant *C. albicans* was >1.28 mg/mL, which was similar to the previous report (E Silva et al., 2018). The MIC of KBN against *C. albicans* was 6.25 mg/mL, and the concentration of borneol in it was only 0.03 mg/mL. It was speculated that borneol had no obvious inhibitory effect on *C. albicans* at this concentration. Therefore, the borneol control group was not established in the series of *C. albicans* trials.

To explore the effect of KBN treating VVC, we used a VVC model mouse induced by FLC-resistant *C. albicans* 901. The estrogen required by this model can promote the adhesion of *C. albicans* in the vagina by increasing the proliferation and thickening of epithelial cells, and also accelerating the growth of *C. albicans* by enhancing glycogen levels in vaginal tissues (Yang et al., 2018). One important indicator for determining antifungal effects is the effects on inhibiting proliferation and adhesion of fungus. KBN significantly reduced the *C. albicans* burden from day 3 onwards. We found that KBN inhibited the growth of *C. albicans* hyphae by Gram stain, and reduced the adhesion of *C. albicans* to the vagina by PAS staining and SEM. The above results support that KBN effectively reduces the growth and adhesion of *C. albicans* in the vagina.

The hyphal formation is an important virulence factor of *C. albicans* associated with biofilm (Shin and Eom, 2019). Hyphae can attach to host cells, damage host tissues, and evade host immune defenses (Rossoni et al., 2018). When the body’s microecological imbalance or immune function is inhibited, *C. albicans* can transform from a yeast state to a hyphae state, and its invasiveness is enhanced (Thomson et al., 2015). During *C. albicans* infecting epithelial cells, adhesion and hyphal formation are linked: adhesion promotes the hyphal formation and hyphal formation stimulates adhesion (Wächtler et al., 2011). In this study, we found that KBN reduced the adhesion of drug-resistant *C. albicans* 901 to abiotic surfaces (Polystyrene) and VK2/E6E7 cells, inhibited the formation of hyphae, and has a protective effect on VK2/E6E7 cells.

The antifungal drugs used in the treatment of VVC mainly include azoles. After azole treatment of VVC, *C. albicans* may become highly drug-resistant, especially to FLC (Marchaim et al., 2012). Multiple molecular mechanisms may operate in FLC-resistance *C. albicans* at the same time. These mechanisms include decreased overexpression and mutations of the *ERG11* gene, alterations in the sterol biosynthesis pathway, and intracellular drug accumulation (Siikala et al., 2010). One of the biofilm resistance mechanisms of *C. albicans* is the slow penetration of the antimicrobial agent through the biofilm (Van Acker et al., 2014). The resistance of biofilm is also closely related to the increase of extracellular matrix (Desai and Mitchell, 2015). In this study, the antifungal effect of KBN was stronger as compared to FLC. KBN inhibited FLC-resistance *C. albicans* 901 biofilm and 12.5 mg/mL of KBN inhibited 75.54% of biofilm formation, while 64 μg/mL of FLC only inhibited 22.23%. KBN significantly reduced the synthesis of ergosterol of FLC-resistant *C. albicans* 901. The expression of ergosterol synthesis-related gene *ERG11* in FLC-resistant *C. albicans* 901 was 1.2 times that of sensitive *C. albicans* 98001, indicating that the overexpression of *ERG11* is one of the FLC-resistance mechanisms of *C. albicans* 901, while KBN down-regulated its overexpression. Interestingly, 64 μg/mL of FLC up-regulated the expression of the FLC-resistant *C. albicans* 901 *ERG11* gene, which was consistent with previous reports (Morais et al., 2021). These results as a whole suggest that the antifungal effect of KBN may reduce the synthesis of ergosterol by inhibiting the gene expression of *ERG11*.

The multidrug resistance in *C. albicans* has previously been proved to be linked to proteins encoded by efflux pump genes *MDR1*, *CDR1*, and *CDR2* (Mukherjee et al., 2003). To further reveal the potential molecular basis of KBN against drug-resistant fungus, we tested the transcription levels of drug resistance genes *MDR1*, *CDR1*, and *CDR2*. In our study, the overexpression of efflux pump genes *MDR1*, *CDR1*, and *CDR2* in FLC-resistant *C. albicans* strain are consistent with previous reports (White et al., 2002; Park and Perlin, 2005; Feng et al., 2018). The results of this experiment showed that KBN significantly down-regulated the expression levels of efflux pump genes *MDR1* and *CDR2* in drug-resistant *C. albicans* 901. The results of this experiment suggested the same antifungal effect of KBN on FLC-sensitive and resistant *C. albicans* might be related to the inhibition of the expression of drug-resistant efflux pump genes *MDR1* and *CDR2*.

A protective immune response is caused by VVC, accompanied by tissue inflammation. Dectin-1 is a type of PRR, which recognizes *β*-glucan in the fungal cell wall (Hardison and Brown, 2012). Recognition of *β*-glucans by Dectin-1 leads to the induction of numerous cellular responses, including respiratory burst, formation of phagosomes, the production of arachidonic acid metabolites, the induction of multiple cytokines, and chemokines (Huysamen and Brown, 2009). After Dectin-1 binds to *β*-glucan, Src family kinases are phosphorylated and the immunoreceptor tyrosine-based activation motif (ITAM)-like motif is activated, which activates Syk; the activated Syk activates CARD9 by the downstream products produced by PLCγ2, forming the CARD9-BCL10-MALT1 complex (Strasser et al., 2012). The CARD9 complex promotes the formation of the IKK complex to enable IκB protein phosphate and ultimately activate NF-κB (Gross et al., 2006), which produces inflammatory cytokines and chemokines (Li et al., 2009). It was previously reported that the levels of pro-inflammatory factors TNF-α, IL-1β, and IL-6 in VVC model mice were elevated (Trinh et al., 2011; Joo et al., 2012). In this study, KBN inhibited the expression of TNF-α, IL-1β, and IL-6 in vaginal tissues, similar to previous reports in vaginal epithelial cells A431 and VK2 (E6/E7) cells (Trinh et al., 2011; Wagner and Johnson, 2012). The Dectin-1/Syk/PLCγ2/Card9/NF-κB signaling pathway was activated in the vaginal tissues in the model group, and KBN significantly inhibited its activation. The above results suggest that KBN can alleviate vaginal inflammation in FLC-resistant VVC model mice by inhibiting the Dectin-1 signaling pathway.

The current study has several limitations. Firstly, KBN is a prescription in TCM with multiple components, and the bioactive components from KBN that relieve VVC remain unclear. Secondly, VVC is also caused by *C. glabrata*, *C. krusei*, *C. tropicalis*, *C. parapsilosis*, etc, and the effect of KBN on FLC-resistant VVC caused by these fungi needs to be further evaluated. Thirdly, the downstream targets of the Dectin-1 signaling pathway are also regulated by other factors, and the in-depth study of downstream mechanisms is crucial for future research. Finally, this study did not assess the change of vaginal microbiota and the effect of KBN on vaginal beneficial bacteria (such as *Lactobacilli*) of VVC mice is unknown. Therefore, we will further research the bioactive components of KBN treating VVC and their interactions, the effect of KBN treating VVC caused by other FLC-resistant fungi, the in-depth downstream mechanisms of the Dectin-1 signaling pathway, and the effect of KBN on vaginal beneficial bacteria in VVC mice in the future.

In conclusion, our study reveals that KBN can ameliorate vaginal inflammation in VVC mice caused by FLC-resistance *C. albicans*. This effect may be related to inhibiting the growth of FLC-resistance *C. albicans* and Dectin-1 activation. Furthermore, our study revealed the potential mechanism of KBN against FLC-resistance *C. albicans*. As the development of new antifungal drugs is progressing slowly and the azole drugs on the market are facing the challenge of resistant fungi, the development of KBN may provide a new treatment option for superficial drug-resistant fungal infections.

## Data Availability

The raw data supporting the conclusions of this article will be made available by the authors, without undue reservation.
